# Investigating the Yanomami malaria outbreak puzzle: surge in mining during Bolsonaro’s government triggered peak in malaria burden

**DOI:** 10.21203/rs.3.rs-4313946/v1

**Published:** 2024-04-29

**Authors:** Daniela de Angeli Dutra, Cor Fontes, Érika Braga, Erin Mordecai

**Affiliations:** Stanford University; Universidade Federal de Mato Grosso; Universidade Federal de Minas Gerais; Stanford University

**Keywords:** Malaria, Amazonia, Brazil, mining, deforestation, land use, Yanomami

## Abstract

The Yanomami, an Indigenous people from the Amazon, confront multifaceted challenges endangering their health and cultural integrity. Of immediate concern is the surge in malaria cases in their territory during Bolsonaro’s government. We investigated the impact of land use on malaria incidence among the Yanomami leveraging satellite imagery and ran difference-in-differences analyses to ask whether the Yanomami suffered disproportionately from malaria when illegal mining was rising in the region (2016–2022). We show a remarkable ~300% rise in malaria from 2016 to 2022 and point to mining as the primary driver of malaria among the Yanomami; when mining increases by 1%, malaria increases by 31%. After mining unfolded, the burden of malaria among the Yanomami was disproportionately higher, up to 15%, than in non-indigenous communities. Our findings underscore the impact of illegal mining on the high malaria burden suffered by the Yanomami and the importance of rainforest conservation and land sovereignty for Indigenous health.

Human malaria is a vector-borne disease caused by *Plasmodium* parasites transmitted by *Anopheles* mosquitoes. This disease is one of the main causes of human infectious disease burden worldwide with hundreds of thousands of deaths and over 200 million cases every year^1^. Malaria is concentrated in sub-Saharan Africa and tropical regions of Asia, Oceania, and Latin America^2^. From the 1980s onwards, the number of malaria cases in Brazil increased as a result of the disorderly occupation of the Amazon region during the military regime^3^. After 2004, control measures based on diagnosis and treatment were intensified and the number of cases was reduced significantly^3^. Afterwards, Brazil faced fluctuations in malaria cases; however, numbers remained significantly lower than the approximately 500 to 600 thousand annual cases observed in the ‘90s^3^. However, in certain regions and communities malaria cases have risen in the last two decades^4^. For instance, the proportion of cases in Indigenous territories and regions subject to artisanal mining increased 300% since 2000^4^. Further, deforestation increases the risk of malaria in Brazil^5–8^, demonstrating that land use alters the local dynamics and risks of malaria. It is important to note that during the presidential administrations of Michel Temer and Jair Bolsonaro (from 2016 to 2022), deforestation and land use change have increased in Brazil^9,10^. These changes could be associated with the recent malaria outbreaks in Indigenous communities, in particular the Yanomami people in North Brazil.

In the Amazon forest, intact forests harbour a lower density of potential vectors of malaria than deforested areas, with no closed-canopy malaria vectors known to occupy the Amazonian forest^6^. Likewise, at ~50% forest cover, the proportion of *Plasmodium*-infected mosquitoes in the Amazon is highest, decreasing with increases or decreases in forest cover. *Nyssorhynchus darlingi* (former *Anopheles darlingi*)is the most important vector and an anthropophilic mosquito in the Amazon. Therefore, anthropogenic land use change (e.g., clear-cutting, logging, mining, agriculture) within or adjacent to forest increases the local abundance of *N. darlingi* as it promotes the expansion of natural breeding habitats and the creation of new breeding habitats on the forest edge^6^. At the same time, mining can drive malaria incidence by three main mechanisms. First, mining increases breeding sites for vectors by creating artificial pools of water^11^. Second, the movement of miners within a region contributes to pathogen dispersal, increasing the probability of local transmission^12^. Finally, the consumption of mercury-contaminated surface and ground water and fish due to mining activities increases the likelihood of contracting malaria, which is potentially mediated by changes in immune response and severe malnutrition induced by mercury exposure^13–15^.

The Bolsonaro administration adopted an environmental policy that eroded forest and Indigenous protections that were previously in place, expanding mining, logging, and agriculture in the Amazon^16^. As a result, the country rolled back environmental regulations and enforcement measures during his administration^17^. One of the most contentious issues has been Bolsonaro’s approach to deforestation. Deforestation rates in the Amazon have surged to alarming levels^16,18^, raising concerns about biodiversity loss, habitat destruction, and climate change^17^. Critics argue that Bolsonaro’s rhetoric and policies have encouraged illegal loggers, miners, and land grabbers, leading to environmental degradation and forest destruction^18^. Those policies particularly affect Indigenous communities, which faced threats over their land rights and protection^16,19^. In addition, the efficiency of Indigenous health services was also diminished by funding and service cuts during Bolsonaro’s government, resulting in a rise in child mortality and suicide^19^. Indigenous territories received multiple requests for commercial exploitation (e.g., mining and logging)^4,20^, potentially threatening the rights and livelihoods of Indigenous peoples.

The Yanomami are an Indigenous people from the Amazon rainforest spanning Brazil and Venezuela (Figure 1). Their territory is located in the north of the Amazonas state and west of Roraima state, on the border of Brazil and Venezuela, and represents the largest Indigenous territory in Brazil. The Yanomami comprise approximately 35,000 to 40,000 people, of which approximately 30,000 live in Brazil according to the Brazilian Ministry of Health database. Many Yanomami people live in isolated Indigenous communities who have no contact with most of Brazilian society. Nonetheless, as of 2022, more than a thousand mining requests had been filed to operate in their territory^20^. During Bolsonaro’s administration, the Yanomami faced persistent threats to their health, culture, and existence^4,15^. In 2023, a major humanitarian crisis triggered by the invasion of 30,000 illegal miners in their territory emerged among these Yanomami people in Brazil with over 300 deaths in 2022. This represents an increase of 50% in deaths compared to previous years. There are four main triggers of this humanitarian crisis: severe malnutrition, poor access to healthcare, mercury contamination due to illegal artisanal gold mining, and high malaria burden^21^.

With this in mind, we aimed to investigate the role of land use change on one of the main triggers of the Yanomami humanitarian crises: the malaria burden. We used panel regression models to identify the causal effects of land use on malaria incidence and difference-in-differences analyses to quantify the effect of increasing mining on the surge of malaria among the Yanomami. Those analyses allowed us to point to illegal mining as the main cause of the exponential increase in malaria cases among Yanomami Indigenous people and confirm that those communities suffered disproportionally from malaria burden compared to non-Indigenous municipalities close to their territory after the increase in illegal mining activities during the Temer and Bolsonaro governments. This evidence supports the role of anti-environmental policies in causing the explosion of malaria cases in Yanomami communities by contributing to increasing mining rates in their territory.

## Results

### Temporal trends in malaria cases

Malaria infections among the Yanomami increased dramatically during the Bolsonaro administration, soaring by 233% from 2018 to 2023 (Figure 2A-B). Looking back at the 2000s, the prevalence of malaria among the Yanomami fluctuated, with cases ranging from 276 in 2003 to a peak of 7,204 in 2010. Subsequently, there was a downward trend from 2011 to 2014, but this progress was reversed from 2015 to 2022, with infections escalating from fewer than 5,000 to nearly 20,000 annually (Figure 2A-B). This trend is not unique to the Yanomami but extends to the municipalities in the vicinity of their territory (Figure 2B). In these areas, a similar pattern emerged: malaria cases increased in 2010, followed by a decline until 2015. However, from 2015 to 2017, there was a resurgence in malaria cases among non-Indigenous municipalities, culminating in a peak of 11,229 cases in 2017. Notably, during the same period (2018 onward) that malaria infections surged in the Yanomami territory, cases dipped from 2017–2019 in non-Indigenous municipalities and then climbed again after 2019; however not as steeply as in the Yanomami (Figure 2B). These trends are not present in all sites; for instance, no change in the number of malaria infections over time was observed in certain sites, both Yanomami and non-Indigenous municipalities (Figure 2C).

### Drivers of malaria in the Yanomami territory

To investigate the drivers of this surge in malaria cases among the Yanomami people, we ran two fixed effects panel regression models to quantify the effect of land use change (i.e., mining and deforestation) on the incidence of malaria (all parasite species) and *Plasmodium falciparum* malaria. Our panel regression model points to mining as the main driver of malaria infections in Yanomami territory (Table 1), with a 1% increase in gold mining increasing the incidence of malaria by 31%. As a result, malaria cases among the Yanomami increased by 12,000 malaria cases (Figure 2B) in response to an increase in mining cover of ~15km² (Figure 3C) from 2016 to 2022. On the other hand, we found that forest cover has a protective effect on the Yanomami people, with an increase of 1% in forest cover decreasing malaria incidence by 0.81%. We observed no association between precipitation or temperature and malaria incidence. Deforestation reduced malaria incidence in the additional models we ran for robustness checks (Sup. Table 5–6) but, unlike the effects of gold mining and forest cover, this effect was not present in our main panel regression model, suggesting that it may be a spurious result of these particular model specifications. When repeating our panel regression analyses using *P. falciparum* incidenceas the response variable, forest cover was the only factor associated (negatively) with malaria incidence; mining had to be removed from this model because of its high correlation with year, so we were unable to address the role of mining for *P. falciparum* only (Table 2).

In addition, we observed increases in mining cover next to the Yanomami aldeias (i.e., Indigenous traditional villages inside the Yanomami territory, Figure 1) from 2020, during Bolsonaro’s administration (Figure 3A-B). Nonetheless, when considering the whole Yanomami Indigenous territory, increases in mining started in 2016, during Michel Temer’s tenure, and then escalated during Bolsonaro’s administration (Figure 3C). No significant mining changes were observed in non-Indigenous municipalities (Figure 1) during that same period (Sup. Figure 1). Further, a clear increase in forest cover occurred next to the Yanomami aldeias from 2019 to 2021 (Figure 3D).

### Impact of mining on malaria incidence among the Yanomami

Due to the large impact of mining on malaria incidence, we asked whether this impact disproportionately affected the Yanomami. To do so, we compared malaria incidence before and after mining escalated between Yanomami and non-Indigenous municipalities using difference-in-differences analyses (for both *P. falciparum* and total malaria incidence). These analyses showed that malaria incidence was disproportionately higher among the Yanomami from 2017, with the largest differences in malaria incidence occurring during Bolsonaro’s tenure from 2019–2021 (Figure 4A-B). It is important to note that our models are particularly conservative as they control for spatial and temporal variation in each unit. This trend is observed when analyzing all malaria cases combined and only *P. falciparum* cases (Figure 4A-B). Our analyses show that yearly malaria incidence among the Yanomami was up to 15% higher when compared to non-Indigenous municipalities, and up to 5% higher when evaluating only *P. falciparum* cases (Sup. Table 1 and 2). The fact that the surge in mining in the Yanomami territory coincides with the largest positive differences in coefficients from the difference-in-differences analyses (Figures 2 and 4) supports the hypothesis that the high malaria burden disproportionately suffered by the Yanomami occurred largely due to the surge in illegal mining in their territory. This result is further supported by the panel regression analyses showing that Yanomami aldeias had higher malaria when experiencing increases in gold mining next to their aldeias, a trend that was not observed in Yanomami aldeias without mining (Table 1).

## Discussion

### Mining and malaria in the Yanomami territory

Vector-borne disease incidence is sensitive to several environmental drivers, including land use, climate, population, and human behaviour^5,22–26^. Land use change (e.g., deforestation and mining) is a known factor that drives malaria incidence and can substantially increase the abundance and human-biting activities of malaria vectors (*Nyssorhynchus darlingi*) in the Amazon Rainforest^5,7,8,27^. We identify mining as the major positive driver of malaria incidence among the Yanomami in recent years, with a 1% increase in gold mining increasing the incidence of malaria by 31% (Table 1). Since the increase in mining activities in 2016, malaria cases in the Yanomami territory have increased from less than 5,000 to close to 17,000 malaria cases (Figure 2B) while mining cover in their territory surged from ~0.04km² to ~15.4km² (Figure 3C). Interestingly, we found that the maintenance of forest cover among these Indigenous communities presents a protective effect against malaria (Tables 1–2). These significant and opposing effects of mining and forest cover on malaria incidence highlight the importance of forest conservation, land sovereignty, and the implementation of policies to prevent illegal mining for the health and livelihood of Indigenous people.

Our research adds nuance to previous studies that suggested an upward trend in malaria cases in the Roraima state, Brazil^28^. Here, we showed that malaria incidence has declined since 2016 in non-Indigenous municipalities, suggesting that our observed upward trend in malaria cases is a result of surges in malaria incidence in Indigenous territories (Figure 2B). Due to the pivotal effect of mining on malaria incidence and the disproportionate malaria burden suffered by the Yanomami highlighted by our study, Brazilian malaria control efforts should prioritize Indigenous territories, particularly those subject to increasing illegal mining activities as those are the most vulnerable to new outbreaks of malaria.

### Recent increase in mining in Amazonian Indigenous territories

It is important to note that the surge in mining started amid a change of government in Brazil, after the impeachment of President Dilma Rousseff in 2015, and significantly increased during the subsequent Temer and Bolsonaro administrations. Trends of increased mining in Indigenous territory from 2019 onward make it clear that Bolsonaro’s anti-environmental policies and rhetoric contributed to the explosion of illegal mining and environmental degradation throughout the country^16,20,29^. Indeed, his anti-environmentalism policy is a central pillar of “Bolsonarism” and its support for global agribusiness^30^. For instance, in 2019 Bolsonaro transferred the responsibility for demarcating Indigenous territories from the National Indigenous People Foundation (FUNAI) to the Ministry of Agriculture, which is influenced by agribusiness interests^31^. Bolsonaro has also expressed on multiple occasions his disdain for Indigenous rights. In 2015, he claimed:

“There are no Indigenous lands where there are no minerals. Gold, tin, and magnesium are found on those lands, especially in the Amazon, the richest region of the world. I do not go with this bulls***t of defending Indigenous lands”^32^.

It is well established that Bolsonaro’s rhetoric encouraged illegal miners and land grabbers to explore and mine Indigenous territories, particularly in the Amazon^10,19,20,33^. In addition, during his administration, multiple requests for help sent to the Federal Prosecutor’s Office, the FUNAI, and the Army in favour of the Yanomami were ignored^4^. Those policies contributed to over 30,000 illegal miners occupying the Yanomami territory in January 2023^34^, highlighting the influence of his government on the malaria burden among these Indigenous people by promoting illegal mining exploration in their territory.

### Forest cover

Interestingly, the surge in mining coincides with increases in forest cover (Figure 3A and D). Artisanal mining in the Amazon is often transitory as illegal miners only exploit surface gold. As a result, the consequent increase in malaria transmission is explained by the focal environmental change (e.g., damming of streams and tanks abandoned due to the scarcity of gold), which favours the creation of habitat for the vector^11^. Wider deforestation emerges when mechanized companies establish themselves in the Amazon forest^35^.

Another important finding of our study is the protective effect of forest cover against malaria: local marginal increases in forest cover led to marginal decreases in malaria (Table 1). This suggests that conservation efforts and the maintenance of forests in Indigenous territories enhance Indigenous health by reducing malaria incidence. In the Amazon, *N. darlingi* is the most important vector and an anthropophilic mosquito. Deforestation increases the local abundance of *N. darlingi* as it promotes the expansion of natural breeding habitats and the creation of new breeding habitats on the forest edge^6^. In addition, the forest cover increased in the last few years next to the Yanomami aldeias (Figure 3D). This may contribute to lowering malaria incidence among Indigenous populations by limiting the creation of new vector habitats and human exposure. In addition, granting full property rights considerably reduces deforestation in Indigenous lands^36^. Thus, the relationship between forest conservation and Indigenous health, evidenced by the protective effect of forest cover against malaria in the Amazon, underscores the critical importance of establishing Indigenous territories and preserving intact ecosystems within Indigenous territories in Brazil to mitigate disease transmission and uphold the well-being of Indigenous communities.

### Limitations

It is important to note that this study has a few limitations. First, it relies on remotely sensed land use data that has some resolution limitations. Satellites may not be able to capture changes in land use on very small scales. In our remote sensing analyses, the resolution of the satellites is 30 meters^37^; therefore, it is possible that some artisanal mining activities and forest loss are not detected in our analyses because their scale is too small. In addition, underreporting of malaria cases in Yanomami populations is common and a known issue as people in these communities often present a predominance of submicroscopic infection. Robortella et al. (2020) have reported that 75 to 80% of malaria infections in Yanomami communities are submicroscopic^38^. In addition, as the Yanomami are a semi-isolated Indigenous people, they do not possess easy and constant access to healthcare and, as a result, malaria diagnosis. Therefore, it is likely the number of malaria cases reported for the Yanomami and the malaria incidence estimated for this group is underestimated. This means that our analyses are likely to report conservative outcomes regarding the impact of mining on malaria incidence in the Yanomami territory. In addition, although we have a mechanistic hypothesis for the disproportionate increase in malaria incidence among the Yanomami (i.e., increasing illegal mining specifically on their territory), the difference-in-differences analyses only pinpoint the time at which the difference between populations occurs without definitively implicating specific mechanisms. Rather, support for the role of mining in driving the disproportionate malaria burden in the Yanomami is bolstered by the panel regression results as well as the context in terms of Bolsonaro’s rhetoric and policy at the time.

## Conclusion

In conclusion, we show that mining is the main driver of the recent surge in malaria in the Yanomami territory. Further, we estimated that an increase of 1% in mining leads to a 31% hike in malaria incidence (Table 1). It is also clear that the disproportionate burden of malaria among the Yanomami coincided with the surge in mining in their territory and got progressively worse as mining increased (Figures 3–4). We showed that malaria cases among the Yanomami were up to 15% higher than in nearby non-Indigenous communities, which is likely a result of surging illegal mining in their territory (see Supplementary Tables 1 and 2). In addition, we highlight that the maintenance of high forest cover protects the health of Indigenous people in the Amazon by protecting against malaria (Table 1–2). These findings make it clear the protection and conservation of forests in Indigenous territories is a pivotal policy to improve Indigenous health and welfare. Moreover, we argue the surges in mining and malaria coincided with Bolsonaro’s pro-mining and anti-Indigenous policies, highlighting the influence of his government on the malaria burden suffered by the Yanomami.

## Methods

### Malaria data

In December 2023, we obtained data on malaria cases in Brazil between 2003 and 2023 from the Brazilian Ministry of Health database (Malaria em áreas indígenas). We selected the variables: year, aldeia (i.e., Indigenous traditional village), parasite species, latitude, longitude, and site of infection. We filtered the data to only include infections among the Yanomami people. Additionally, we extracted data on malaria infections from 2007 to 2023 in nearby municipalities in the vicinity of the Yanomami territory in the Amazonas and Roraima states from the Brazilian Ministry of Health database (Malaria nas regiões amazônica e extra-amazônica) in January 2024. Cases assigned to Indigenous people were removed from this second dataset. To ensure that we only included data from municipalities close to the Yanomami territory, we filtered the data to consider malaria infections from sites with a latitude of −2 degrees or higher. Data on the location of each aldeia was obtained based on the location data provided by the Ministry of Health database.

### Remotely sensed land use variables

In total, 64 sites notified malaria cases among Yanomami people (Figure 1) and 20 surrounding municipalities notified cases of malaria among non-Indigenous populations. We used the Google Earth Engine platform to extract annual data on forest, farm, and mining cover from MapBiomas Brasil (https://brasil.mapbiomas.org/)^37^ for each diagnosis centre from 2003 or 2007 to 2021. Cover data was extracted as the land use cover in a 10 km² polygon around the geographic coordinates associated with the diagnosed location of malaria infections. Annual deforestation and mining changes were calculated by comparing the cover with the previous year.

### Population data

Population count data was acquired from two distinct sources. Data for each municipality’s population for each year was obtained from LandScan (https://landscan.ornl.gov/) using the Google Earth Engine platform to extract the data for each 10 km² polygon around the geographic coordinates associated with the diagnosed location of malaria infections. For the Yanomami people, population size at each polo base (i.e., territory subregion) was obtained from the Ministry of Health database due to the lack of data from LandScan for Indigenous communities.

### Climatic variables

Average annual temperature at 2m from the ground and total annual precipitation were estimated for all site 10 km² polygons, in both Yanomami territory and municipalities. Both temperature and precipitation variables were extracted data from the high-resolution (0.05-degree resolution) datasets of the Climate Research Unit database (https://crudata.uea.ac.uk/cru/data/hrg/)^39^. To calculate the average annual temperature at 2m for each pixel, monthly values were averaged. For total annual precipitation in each pixel, monthly precipitation values were summed. Afterwards, since we extracted data for the entire polygon, we calculated the mean value of each observation within the polygon. Therefore, the temperature at 2m represents the mean temperature at 2m for each polygon during the whole year while total annual precipitation represents the mean total precipitation observed within each polygon.

### Statistical analyses

For all statistical analyses, we used the package “fixest”^40^ in R version 4.3^41^. A fixed effects panel regression model was run to evaluate the effects of land use and climate variables on the incidence of malaria infections among the Yanomami people using the function “fepois”. Here, we only used the data for the 64 Yanomami sites. Our panel data presents a hierarchical structure with units of our analysis being each site (aldeia) in each year. We consider the incidence of malaria per site and year (i.e., total number of malaria cases divided by population for each year and site) as our response variable. The annual forest cover, change in mining, and deforestation, along with the annual average temperature at 2m and total precipitation were used as explanatory variables in our model. We also included unit and year fixed effects to control for time-invariant geographic differences and shared temporal trends in malaria, respectively. This ensures that our estimates of marginal effects of the explanatory variables of interest are not confounded by cross-sectional or shared temporal variation; in effect, this model compares each unit to itself over time to obtain estimated effects of covariates. Due to the right-skewed distribution of our data, we employed Poisson distribution in the panel regression model. Mining changes were removed from panel regression evaluating *P. falciparum* incidence due to the high correlation with year. The formula describing the model is as follows:

(1) $$Y_{it}=\sum \;\lambda_{i}+\mu_{t}+\theta X_{it}+\varepsilon_{it}$$

Where:

- $Y_{it}$ is our response variable, malaria incidence in each aldeia i and year t.
- $\lambda_{i}$ and $\mu_{t}$ are the fixed effects associated with each spatial and temporal unit (i.e.,. aldeia i and year t).
- i and t represent the indices for aldeias (units) and years,. Respectively.
- θ are the coefficients associatted with the control variables $X_{it}$ {i.e., fore.st covet,. change in mining, deforestation, the average temperature at 2m, and total precipitation).
- $\varepsilon_{it}$ represents. the error term.

In addition, after confirming support for mining as the main driver of malaria incidence among the Yanomami in the panel regression, we investigated whether the Yanomami people were disproportionally affected by malaria after the surge in mining in the region in 2016, compared to non-Indigenous groups (Figure 3C). To do so, using the function “feols” we ran a difference-in-differences analysis comparing the malaria incidence in the Yanomami territory with the surrounding non-Indigenous municipalities with notified cases, before and after the increase in mining. Here, we ran an ordinary least squares (OLS) model combining the Yanomami and municipality data (malaria incidence, climatic variables, and fixed effects). This data comprised the years 2008 to 2021. Again, the incidence of malaria infections per unit-year was used as the response variable. Since the goal of this model was to evaluate the impact of the increase in mining during the Temer and Bolsonaro governments on malaria incidence, we compared Yanomami sites before and after 2015 (the last year before mining increased in Yanomami territory) against non-Indigenous municipality data at the same period. The interaction between Yanomami and the years of Temer and Bolsonaro governments were employed as interacting explanatory variables; the interaction coefficient effect for each unit-year is the predictor of interest used for difference-in-differences estimates. In addition, the model included fixed effects for unit (aldeia or municipality) and year as well as the average temperature at 2m and total annual precipitation as control variables. The formula describing the model is as follows:

(2) $$Y_{it}=\sum_{\begin{array}{l} r\in \left\{2008,\ldots ,2021\right\}\\ \;r\neq 2015 \end{array}}\;\beta_{t}{\left(Yanomami\;\times \;Year\;\right)}_{i}x\;1\left\{t=r\right\}+\lambda_{i}+\mu_{t}+\theta X_{it}+\varepsilon_{it}$$

Where:

- $Y_{it}$ is our response variable, malaria incidence in each aldeia or municipality i and year t.
- Yanomami is a binary variable indicating whether the malaria incidence value is from the Yanomami people or non-Indigenous people.
- Year is a variable indicating the year of each observation, where the reference level is 2015 (the year before mining increased).
- $\beta_{t}$ is the interaction between year and Yanomami people, i.e., the difference-in-differences estimate for a given year t, where the years after 2015 are the coefficients of interest.
- $\lambda_{i}$ and $\mu_{t}$ are the fixed effects associated with each spaitial and temporal unit (i.e., aldeia or municipality i and year t).
- i and t represent the indexes for aldeias and municipalities (units) and years.
- θ are the coefficients associated with the control variables $X_{it}$ (i.e., the average temperature at 2m and total precipitation).
- $\varepsilon_{it}$ represents the error term.

Both analyses were repeated using only *P. falciparum* incidence, the parasite species associated with higher morbidity and virulence, as response variables. All models (panel and DID) were weighted for population size in each unit in each given year.

For robustness checks, we ran two additional models for both total malaria incidence and *P. falciparum* incidence. The first model removed climate variables and the second model removed forest cover. The effects found for mining and forest cover on total malaria and *P. falciparum* incidence presented similar magnitudes in both additional models (see Supplementary Tables 3–6). Further, we also ran a placebo permutation test to confirm the robustness of our difference-in-differences analysis. For this, we randomly assigned the “Yanomami” variable, shuffling the treatment group. Then, we repeated our analysis 50 times with this shuffled dataset. As expected, no difference in incidence was found pre- and post-treatment when running the models with the permuted dataset (See Sup. Figure 2).

## Figures and Tables

**Table 1:** Panel regression results for total malaria incidence. Estimates, standard errors, *t* and *p* values of the impact of forest cover, deforestation, mining changes, the average annual temperature at 2m, and total annual precipitation on the incidence of malaria in Yanomami communities.

	Estimates	Standard error	t-value	p-value
***Forest cover***	**−0.811**	**0.140**	**−5.787**	**<0.001**
*Deforestation*	−0.168	0.048	−3.496	<0.001
***Mining change***	**30.948**	**0.348**	**88.759**	**<0.001**
*Annual temp.*	−0.384	0.407	−0.942	0.346
*Annual prec.*	<0.001	0.872	<0.001	0.999

**Table 2:** Panel regression results for *P. falciparum* incidence. Estimates, standard errors, *t* and *p* values of the impact of forest cover, deforestation, the average annual temperature at 2m, and total annual precipitation on the incidence of *P. falciparum* infection in Yanomami communities. The mining changes variable was removed from the model due to the high correlation with year.

	Estimates	Standard error	t-value	p-value
***Forest cover***	**−0.499**	**0.152**	**−3.274**	**0.001**
*Deforestation*	−0.090	0.060	−1.485	0.137
*Annual temp.*	−0.259	0.427	−0.607	0.543
*Annual prec.*	0.285	1.030	0.277	0.781

## Data Availability

All data and programming codes used in this research will be deposited in Dryad and available as supplementary material after publication acceptance.
